# Anti-Phospholipid Antibodies and Coronavirus Disease 2019: Vaccination Does Not Trigger Early Autoantibody Production in Healthcare Workers

**DOI:** 10.3389/fimmu.2022.930074

**Published:** 2022-07-15

**Authors:** Maria Orietta Borghi, Mauro Bombaci, Caterina Bodio, Paola Adele Lonati, Andrea Gobbini, Mariangela Lorenzo, Erminio Torresani, Antonella Dubini, Ilaria Bulgarelli, Francesca Solari, Francesca Pregnolato, Alessandra Bandera, Andrea Gori, Gianfranco Parati, Sergio Abrignani, Renata Grifantini, Pier Luigi Meroni

**Affiliations:** ^1^ IRCCS Istituto Auxologico Italiano, Immunorheumatology Research Laboratory, Milan, Italy; ^2^ Department of Clinical Sciences and Community Health, University of Milan, Milan, Italy; ^3^ Istituto Nazionale Genetica Molecolare, Padiglione Romeo ed Enrica Invernizzi, Milan, Italy; ^4^ Infectious Diseases Unit, Foundation IRCCS Ca’ Granda Ospedale Maggiore Policlinico, Milan, Italy; ^5^ Centre for Multidisciplinary Research in Health Science (MACH), University of Milan, Milan, Italy; ^6^ Department of Medicine and Surgery, University of Milano-Bicocca, Milan, Italy

**Keywords:** anti-phospholipid antibodies, SARS-CoV-2 vaccination, autoimmunity, autoantibodies, COVID-19

## Abstract

A molecular mimicry between severe acute respiratory syndrome coronavirus 2 (SARS-CoV-2) and human proteins supports the possibility that autoimmunity takes place during coronavirus disease 2019 (COVID-19) contributing to tissue damage. For example, anti-phospholipid antibodies (aPL) have been reported in COVID-19 as a result of such mimicry and thought to contribute to the immunothrombosis characteristic of the disease. Consistently, active immunization with the virus spike protein may elicit the production of cross-reactive autoantibodies, including aPL. We prospectively looked at the aPL production in healthcare workers vaccinated with RNA- (BNT162b2, n. 100) or adenovirus-based vaccines (ChAdOx1, n. 50). Anti-cardiolipin, anti-beta2 glycoprotein I, anti-phosphatidylserine/prothrombin immunoglobulin G (IgG), IgA, and IgM before and after vaccination were investigated. Anti-platelet factor 4 immunoglobulins were also investigated as autoantibodies associated with COVID-19 vaccination. Additional organ (anti-thyroid) and non-organ (anti-nuclear) autoantibodies and IgG against human proteome were tested as further post-vaccination autoimmunity markers. The antibodies were tested one or three months after the first injection of ChAdOx1 and BNT162b2, respectively; a 12-month clinical follow-up was also performed. Vaccination occasionally induced low titers of aPL and other autoantibodies but did not affect the titer of pre-existing autoantibodies. No significant reactivities against a microarray of approximately 20,000 human proteins were found in a subgroup of ChAdOx1-vaccinees. Consistently, we did not record any clinical manifestation theoretically associated with an underlying autoimmune disorder. The data obtained after the vaccination (two doses for the RNA-based and one dose for the adenovirus-based vaccines), and the clinical follow-up are not supporting the occurrence of an early autoimmune response in this cohort of healthcare workers.

## Introduction

Severe acute respiratory syndrome coronavirus 2 (SARS-CoV-2) and, in particular, the Spike protein share common amino acid linear sequences and some conformational structures with human proteins (1, 2). Consistently, an autoimmune signature was suggested to take place in Coronavirus disease 2019 (COVID-19) because of the molecular mimicry phenomenon and the deregulated immune response caused by the virus itself (3–8). Autoantibodies against self-antigens, in particular, anti-phospholipid antibodies (aPL), were reported in COVID-19 patients and their pathogenic role in the disease was discussed (9–12).

If the molecular mimicry with the virus, and in particular with the Spike protein, is playing a role in COVID-19, then the SARS-CoV-2 vaccines could potentially trigger autoimmune responses as well. Consistent with this hypothesis are the induction of the vaccine-induced thrombocytopenic thrombotic (VITT) syndrome and anecdotal cases of *de novo* autoimmune diseases triggered by the anti-SARS-CoV-2 vaccination (13–15). However, the occurrence of autoantibodies before and after vaccination in healthy subjects has not been investigated, and very few studies have addressed whether the vaccines affect autoantibodies already present in autoimmune patients (16, 17). On the other hand, this aspect is becoming of paramount importance because of the large number of vaccinated people and the need for repeated booster injections.

With this in mind, we planned a prospective study to look at the production of autoimmune antibodies in healthcare workers vaccinated with RNA- or adenovirus-based vaccines. Anti-phospholipid antibodies and additional organ and non-organ specific autoantibodies were tested before and after the vaccination. To further investigate whether active immunization may trigger autoimmune responses, the autoantibodies against an array of human proteins before and after vaccination were also investigated in a subgroup of ChAdOx1-vaccinees and the clinical follow-up was planned for all the vaccinated subjects up to 12 months after the first dose of the vaccines. The data obtained after the vaccination (two doses for the RNA-based and one dose for the adenovirus-based vaccines) and the clinical follow-up are apparently ruling out the occurrence of early autoimmune response in this cohort of healthcare workers.

## Materials and Methods

### Vaccinated Subjects

One hundred healthcare workers vaccinated with BNT162b2 (Comirnaty) and 50 with ChAdOx1 (Vaxzevria) between January and February 2021 were included in the study. The inclusion criteria were age >18 years and availability for serial blood samples collection. The exclusion criteria were pregnancy, breastfeeding, autoimmune diseases, other vaccinations in the past 6 months (with the exception of the flu vaccination), and severe adverse effects to previous vaccinations. The demographic characteristics of the vaccinated subjects are reported in **Supplementary Table 1**. Two subjects, vaccinated with BNT162b2, were known to have subclinical autoimmune thyroiditis without any treatment. Fifteen of 100 subjects vaccinated with BNT162b2 and five of 50 subjects vaccinated with ChAdOx1 had mild or asymptomatic COVID-19 confirmed by means of a positive reverse transcription polymerase chain reaction (RT-PCR) for SARS-CoV-2. The infections were documented before the vaccination.

Adverse side effects as defined by Polack et al. (18) or any clinical manifestation potentially correlated with vaccination were also recorded for all the investigated subjects (**Supplementary Table 2**). The follow-up of the vaccinated subjects was carried out for 12 months starting from the first injection of the vaccine.

The Ethics Committee at Istituto Auxologico Italiano approved the study (08–01–2021).

Vaxzevria samples were collected at IRCCS Ca’ Granda Ospedale Maggiore Policlinico in Milan (Polimmune-COVID: approved by Institutional Review Board Milano Area 2 and by the National Ethical Committee of Ospedale Spallanzani, #331_2020 and #665_2020). All the subjects gave their informed consent and their privacy rights are observed.

### Anti–Severe Acute Respiratory Syndrome Coronavirus 2 Serological Profile

The presence of total Ig against the SARS-CoV-2 nucleocapsid and the spike protein RBD was detected by a chemiluminescence solid-phase assay according to the manufacturer’s instructions (Roche, Milan, Italy). Values ≥ 0.80 U/ml were considered positive for anti-RBD Ig; anti-N Ig levels were expressed as a cutoff index (ICO ≥ 1).

Anti–SARS-CoV-2 antibodies were detected immediately before vaccine administration (T0) and 1 month or 3 months after the first injection of ChAdOx1 or BNT162b2, respectively (one dose for the adenovirus-based and two doses for the RNA-vaccine) (T1).

### Detection of Diagnostic Autoantibodies

An autoantibody profile including both organ- and non-organ specific antibodies was investigated in serum samples collected at T0 and T1 as stated above.

Anti-cardiolipin (aCL) and anti-beta2 glycoprotein I (β_2_GPI) immunoglobulin G (IgG), IgM, and IgA were measured by in-house enzyme-linked immunosorbent assay (ELISA), as previously described (17); anti-phosphatidylserine/prothrombin (PS/PT) IgG and IgM were detected by commercial ELISA (Inova Diagnostics, S. Diego, CA, USA), according to the manufacturer’s protocol.

Antinuclear IgG antibodies (ANA) were detected by a commercial chemiluminescence solid-phase assay (CTD screen PLUS; QUANTA Flash, Inova Diagnostics, San Diego, CA, USA) that detects the following cell autoantigens: dsDNA, RNP, Sm, Scl-70, Jo-1, Ro52, Ro60, SS-B, Centromere, RNA Pol III, Pm/Scl, Mi-2, PCNA, Th/To, Ku, ribosomal-P. The assay was performed according to the manufacturer’s instructions (19). The use of a solid-phase assay for ANA as a screening assay for systemic lupus erythematosus classification and for the diagnosis of other ANA-associated rheumatic diseases has been recently suggested and supported by recommendations of international scientific societies (20–22).

Anti-platelet factor (PF) 4 antibodies IgG/IgA/IgM were tested by ELISA (Immucor, Solihull, UK), as reported (17).

Antibodies against thyroid-stimulating hormone (TSH) receptor (TSH-R), thyroglobulin (TG), and thyroid myeloperoxidase (TPO) were detected by a commercial chemiluminescence solid-phase assay (Phadia AB, Uppsala, Sweden) and expressed in IU/ml (TG, TPO) or IU/l (TSH-R), according to the manufacturer’s instructions.

### HuProt Arrays and Serum Profiling Assays

The human proteome microarrays (HuProt) arrays were manufactured by CDI LABS (CDI Laboratories, Inc., Mayaguez, PR, USA). Each HuProt array v4.0 consists of >20,000 human proteins, most full length, and representing >16,000 human protein-coding genes, covering about 80% of the human proteome. The arrays also contained IgG spotted at different concentration (100, 25, 6.25, and 1.56 ng/μl). Incubation was performed according to the manufacturer’s instructions. Briefly, the arrays were blocked using blocking buffer (2% BSA in phosphate-buffered saline [PBS] buffer with 0.05% Tween 20) at 4°C overnight. Then, each serum sample was diluted 1:500 in blocking buffer and incubated at room temperature (RT) for 2h with gentle rocking. After 3 × 10 min washes with TPBS (PBS buffer with 0.05% Tween 20), the arrays were incubated for 2h at RT with Alexa 647–conjugated goat anti-human IgG (Thermo Fisher Scientific, Walthman, MA, USA) (1:800 in blocking buffer) in the dark with gentle rocking. The arrays were then washed twice in TPBS, twice in 0.1× PBS, and finally once in milliQ sterile water. The slides were finally dried at 30°C under nitrogen and scanned using a ScanArray Gx PLUS (Perkin Elmer, Shelton, CT, USA). Images (16 bit) were generated with the ScanArray™ software at a resolution of 10 μm per pixel and analyzed using ImaGene 9.0 software (Biodiscovery Inc., Hawthorne, CA, USA). A 635-nm laser was used to excite the Alexa-647 dye.

HuProt arrays were used to analyze the reactivity of sera from 10 individuals collected pre and post-ChAdOx1 vaccination, five additional individuals only post-ChAdOx1vaccination, and 10 age and sex-matched blood donors (healthy controls, HC sera) collected before the COVID-19 pandemic and retrieved from the archives of IRCCS Istituto Auxologico Italiano and Ospedale Maggiore Policlinico, Transfusional Unit, Milan, Italy. These HC samples were tested on the proteome array in order to identify occasional reactivities spontaneously arising in healthy individuals.

### HuProt Arrays Data Analysis

For each sample, the raw mean fluorescence intensity (MFI) values of each spot were measured, signal-to-local-background ratios were calculated using ImaGene, and spot morphology and deviation from the expected spot position were considered using the default ImaGene settings. For each sample, the background-subtracted MFI of replicated spots was determined. For each protein, a coefficient of variation (CV%) was calculated on replicate spots, for intra-assay reproducibility. Each protein was checked for displaying a CV% correlated to its MFI on the basis of standard IgG curves. If the CV% value was not within the expected range the protein was not considered for further analysis. In order to compare the autoreactivities from independent experiments, the MFI values were normalized based on the IgG curve spotted on each slide (**Supplementary Figure 1**). On the basis of this analysis, we also derived the concentration of autoantigen-specific IgG in ng/µl (**Supplementary Figure 1**). Autoantigens were identified using two different comparisons: (1) 15 post-vaccination versus 10 pre-vaccination sera, using as threshold a fold change of MFI values > 2.5; 2) 15 post-vaccination versus 10 HC samples. In the latter analysis, we selected autoantigens matching these multi-criteria: (i) not recognized by any of HC sera, (ii) having fold change > 2.5 in at least one vaccinated individual, and (iii) having concentration >30 ng/µl (this concentration threshold was established based on the protein reactivity distribution of ChAdOx1 towards HC sera, as shown in **Supplementary Figure 3**).

### Statistical Analysis

The demographic and clinical characteristics of participants were summarized using descriptive statistics. Normalized data and statistical analysis of proteins spotted into the HuProt Array were analyzed by R. Differential analysis between pre-vaccine and post-vaccine were performed with Limma package and *p*-values were corrected by Benjamini-Hochberg procedure.

## Results

### Response to Vaccination of Healthcare Workers

All the vaccinated subjects displayed Ig values against the SARS-CoV-2 spike protein RBD higher than the established cutoff. Twenty of 150 subjects reported previous history of SARS-CoV-2 infection and displayed Ig against the virus nucleocapsid as well.

The percentages of non-severe side effects after the vaccinations were comparable to those reported in the literature (18) and are shown in **Supplementary Table 2**; no severe side effects were reported.

All the enrolled subjects have been followed up for 12 months starting from the first injection of the two vaccines.

### Identification of a Panel of Diagnostic Autoantibodies in Vaccinated Healthcare Workers

**Figure 1:**
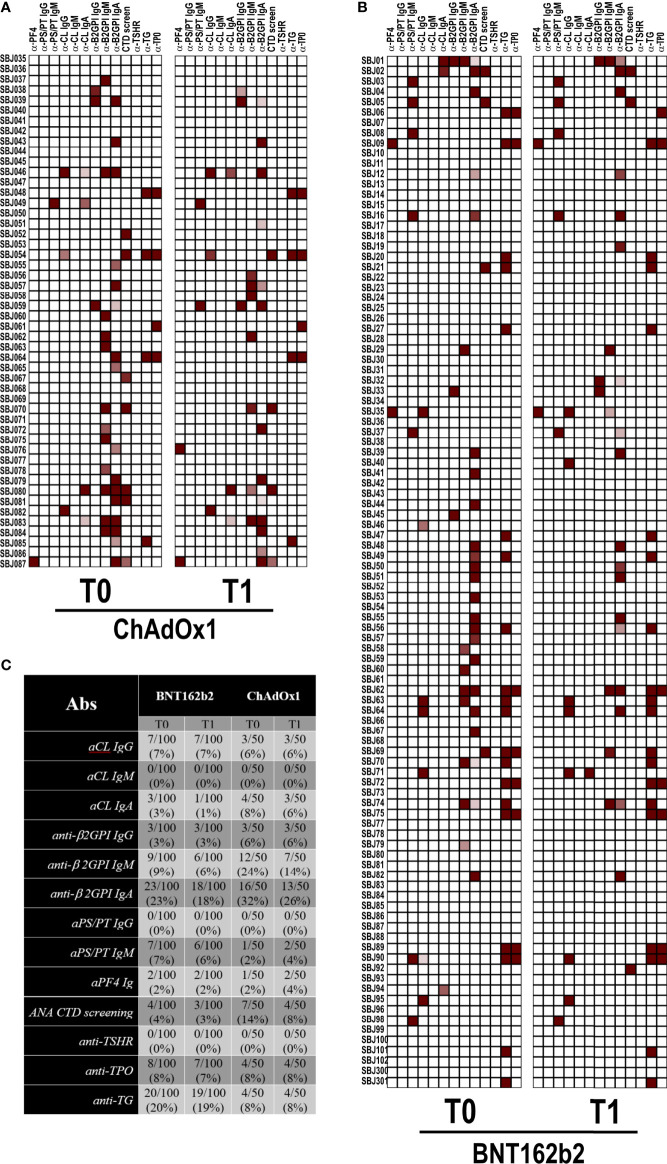
Autoantibody reactivity pre- and post-vaccination by BNT162b2 or ChAdOX1. Heatmap showing serum IgG reactivity of individuals vaccinated with **(A)** ChAdOX1 or **(B)** BNT162b2 to the indicated autoantigens. In red are depicted the reactive subjects (arranged in rows) for each autoantigen (in column). **(C)** Frequencies of reactivity for each autoantigen.

Since aPL and anti-PF4 antibodies were reported in few COVID-19 patients (12, 17) and anti-PF4 antibodies have been associated with the development of VITT after ChAdOx1 vaccination (13–15), we detected the abovementioned autoantibodies in our cohort of vaccinated healthcare workers. Anti-nuclear antibodies were also occasionally reported, and there is evidence of thyroid involvement in COVID-19 patients (9, 23, 24). In keeping with this finding, we tested our vaccinated subjects for an ANA screening assay and anti–TSH-R, anti-TG, and anti-TPO antibodies as further clues of an autoimmune signature. As shown in the heatmaps of **Figure 1**, only occasional positivity was found and the percentages of positivity were very small or the search resulted negative. In particular, we did not detect any new autoantibody positivity or increased autoantibody titers after both vaccines. The positive results were found at low titer with weak clinical diagnostic/prognostic value. This was particularly true for IgA anti-β2GPI positivity (25, 26).

### Identification of Autoantigens Recognized by Sera of Subjects Pre- and Post-Vaccination

**Figure 2:**
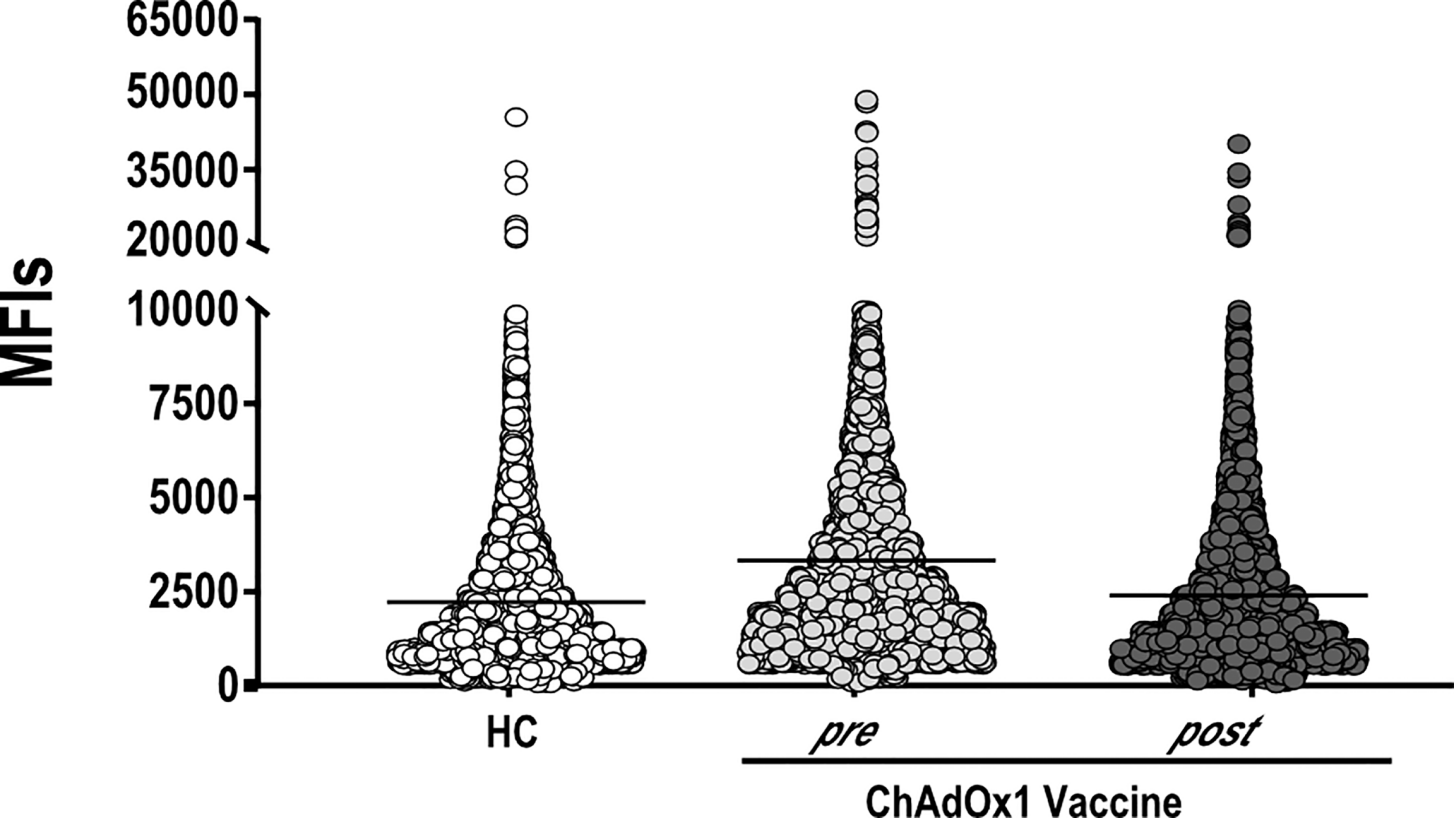
ChAdOx1 vaccination does not alter the general autoimmunoreactivity. Mean Fluorescence Intensities values (MFIs) of all proteins spotted on the HuProt array probed with sera of ChAdOx1 individuals at pre- (*n =* 10) and post-vaccination (*n =* 15) and 10 Healthy Controls (HC). Each dot represents the MFI of a single protein within the groups of sera reported on the *x*-axis.

An important question is whether vaccination may alter autoreactivity in healthy individuals. Keeping this in mind, we evaluated the autoantigen reactivity of sera collected from 15 individuals who received ChAdOx1 against a broad spectrum of human proteins. For 10 of the 15 individuals, we tested both the pre-vaccination and the post-vaccination serum samples. For the five remaining individuals, only the post-vaccination sample was used. The overall autoreactivity pattern of ChAdOx1 vaccinées toward self-proteins, based on the distribution profile of the MFI values for each protein represented on the array, was comparable to that of pre-vaccine and of non-vaccinated HC sera collected in the pre-COVID-19 period (**Figure 2**). None of the antigens showed a significant autoreactivity profile in ChAdOx1 compared to HC group (**Supplementary Figure 2**).

**Figure 3:**
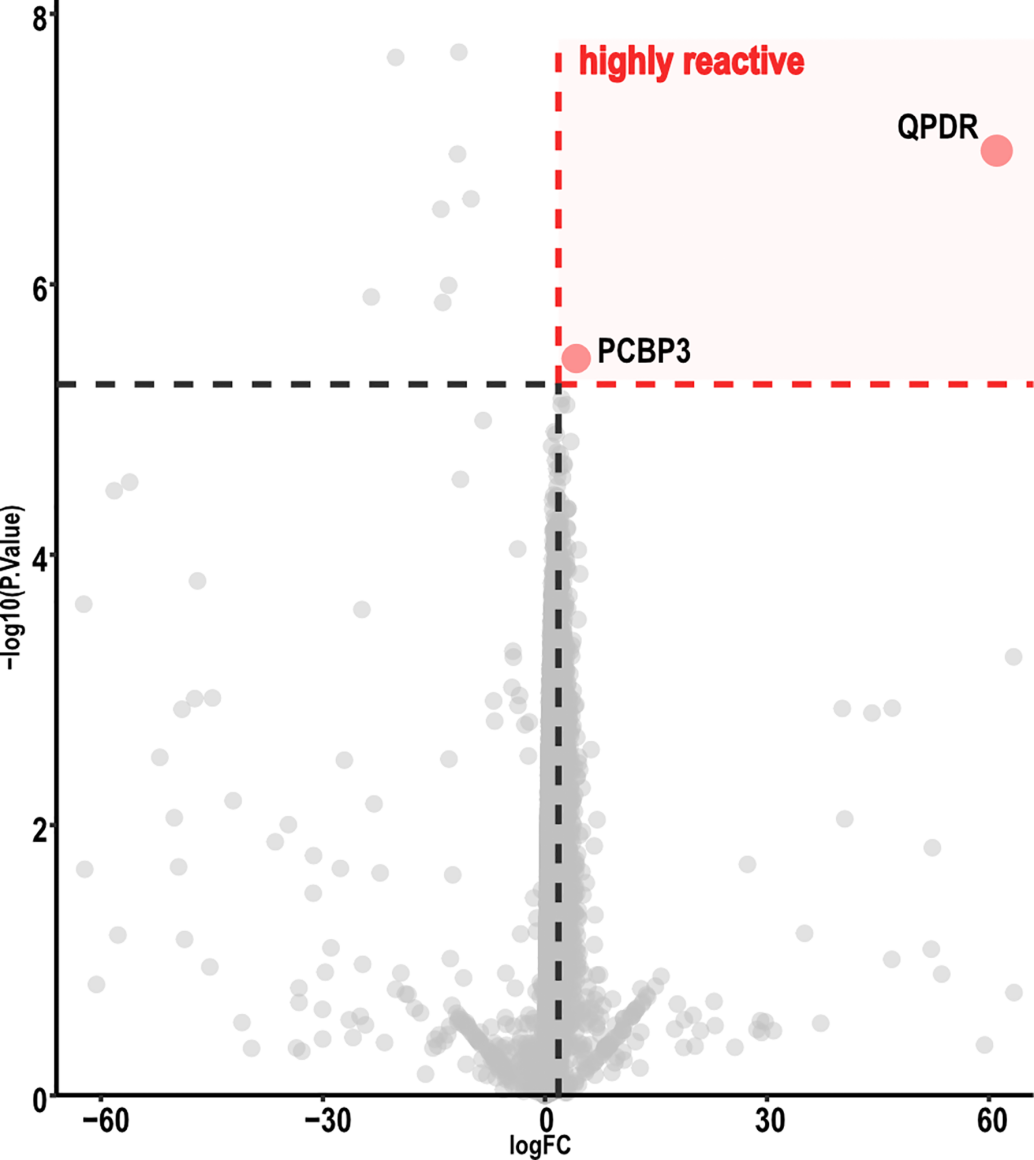
Volcano map analysis of ChAdOx1 samples. Volcano plot representing FC versus *P*-values of each protein displayed only two proteins highly reactive with ChAdOx1 serum samples. Graphic representation focuses from −60 to 60 in a log FC range. Thresholds were established by analysis of the FC (≥ 2.5) and adjusted *P*-value (< 0.01). ChAdOx1 was compared by R package Limma; Benjamini, as well as Hochberg procedure, was used to adjust *P*-values.

Volcano-plot analysis revealed only two highly reactive autoantigens (**Figure 3**), namely, Quinoid Dihydropteridine Reductase (QDPR) and Poly(rC)-binding protein 3 (PCBP3). Frequency analysis indicated that QDPR showed the highest autoreactivity, being recognized by all the subjects (including the HC), whereas PCBP3 was recognized only by one subject after vaccination.

**Figure 4:**
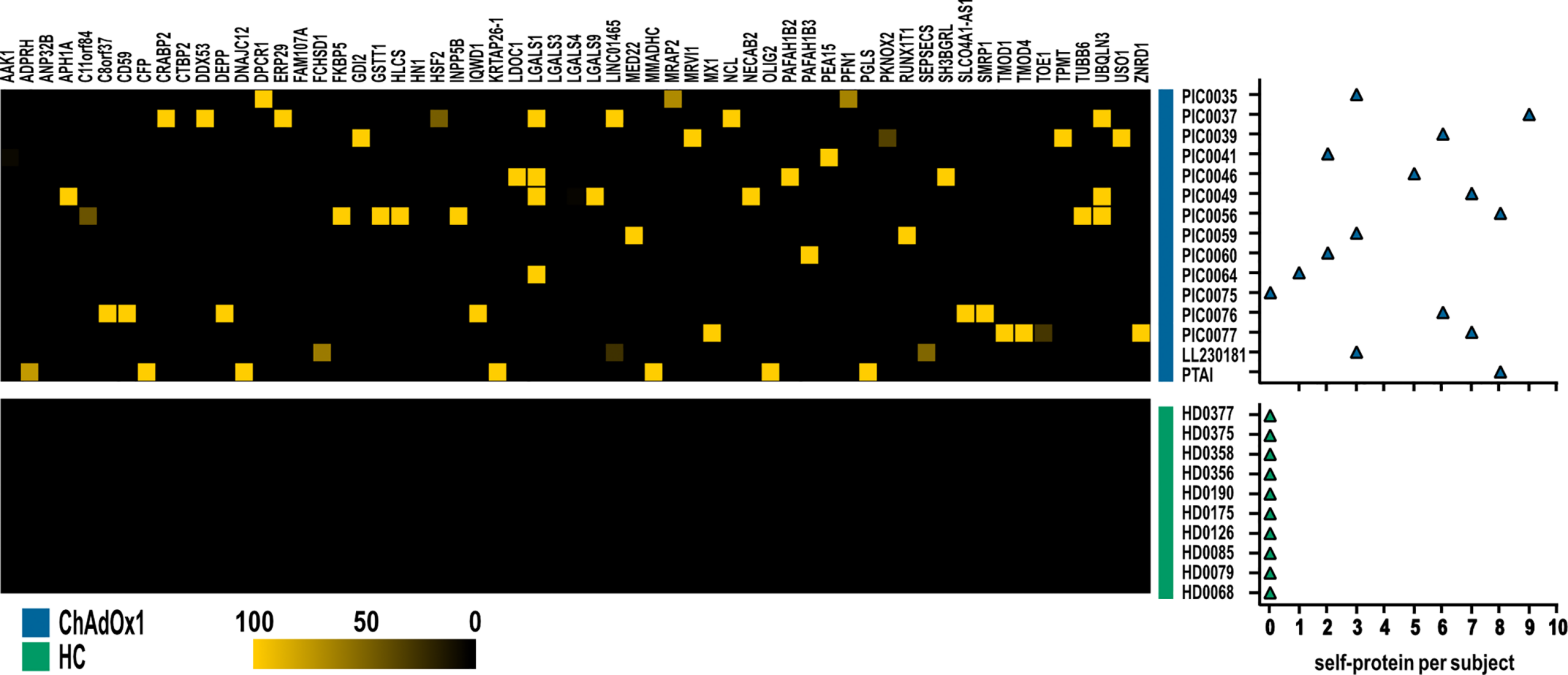
Autoantigens specifically recognized by at least one subject in ChAdOx1 group as opposed to HC. Heatmap depicting IgG reactivities of ChAdOx1vaccinated subject (*n =* 15, in blue) collected post-vaccination, and healthy donors (HC; *n =* 10, in green), arranged in rows, for the 59 antigens (in column). Yellow indicates positive immune reactivity (>30 ng/μl), and black indicates low or no immune-reactivity (<30 ng/μl). The count of the self-proteins recognized by ChAdOx1 vaccinated subjects and HC (blue and green, respectively) is shown in the right panel. Counts were based on antibodies that were present at levels ≥30 ng/μl.

Then, we further investigated specific autoantibodies arising with low frequency upon ChAdOx1 vaccination as detailed in the paragraph 2.5, Materials and Methods section. In this way, we excluded occasional reactivities spontaneously arising in healthy individuals, not specifically ascribable to COVID-19, and we identified 59 proteins, showing a scattered immune-reactivity among the vaccinated individuals (**Figure 4** and **Supplementary Table 3**).

Overall, our analysis showed that ChAdOx1 vaccination does not alter the autoreactivity of vaccinated individuals and only sporadically induces autoantibodies. The subjects who resulted positive for these antibodies have been asymptomatic for the whole clinical follow-up.

## Discussion

Molecular mimicry between the virus and self-antigens was suggested to trigger the production of autoantibodies in COVID-19 and potentially after SARS-CoV-2 vaccination as well. A lot of attention has been paid to aPL in COVID-19 as a prototype of autoantibodies potentially contributing to the thrombophilic state that characterizes the most severe forms of COVID-19. So, we firstly investigated whether the active immunization with both RNA- and adenovirus-based vaccines against SARS-CoV-2 induces the production of aPL as markers of a pro-thrombotic phenotype in asymptomatic healthcare workers. We also investigated anti-PF4 Ig as autoantibody markers of the thrombotic thrombocytopenic purpura that has been recently described after the adenovirus-based anti–SARS-CoV-2 vaccine. Additional non-organ specific, such as ANA, and organ-specific anti-thyroid autoantibodies were evaluated before and after the vaccination, since all of them have been reported in COVID-19 as markers of an unwanted autoimmune response. Our results support the safety of the vaccination from this point of view, since all these autoantibodies have not been developed early after the first injection. Moreover, there is no evidence that the vaccination may affect the titer of pre-existing autoantibodies occasionally detectable in the asymptomatic subjects before the vaccination. In particular, this is true for aPL in agreement with similar data reported in patients suffering from the anti-phospholipid syndrome that received the vaccination against SARS-CoV-2 (17). Consistently, we did not record any clinical manifestation theoretically associated with the development of an underlying autoimmune disorder. This finding is well supported by the data from the vaccinated subjects investigated before and after the complete vaccination with BNT162b2 (two doses). Although the subjects that received ChAdOx1 were tested before and after the first dose only, the absence of newly developed autoantibodies or of clinical manifestations 1 month after the first vaccine injection may support the same conclusion. In fact, the best described autoimmune adverse reaction after ChAdOx1 is the VITT associated with the appearance of anti-PF4 Ig usually a few days after the first administration of ChAdOx1. This suggests that a single immunization can be sufficient for triggering an autoimmune response and the clinical syndrome (13–15). Although some of these autoantibodies resulted positive at low titer, the lack of titer modification before and after the vaccination and the absence of clinical or laboratory signs of autoimmune disorders do support the occurrence of the so-called “innocent” antibodies, as we recently described (12, 17).

We also found few reactivities of the sera from the same cohort against a human protein microarray representing approximately 20,000 human proteins. Since adenovirus-based vaccines has the highest prevalence of severe adverse events associated with definitive autoantibody immunity compared to mRNA vaccines (13–15), we investigated the autoreactivity against the proteome microarray in subjects that received the ChAdOx1 vaccine only. We did not observe any significant alteration of the overall reactivity before and after vaccination or comparing the vaccinated subjects to the non-vaccinated pre-Covid-19 HCs. Indeed, PCBP3 and QDPR 1 were the only two antigens against which we found a high reactivity. However, PCBP3 was recognized by only one subject post-vaccination, whereas QDPR was recognized by all the individuals, including the healthy donors, thereby unlikely being specifically induced by the ChAdOx1 vaccine. In addition, when comparing ChAdOx1 vaccinated versus non-vaccinated healthy individuals, we identified 59 proteins that were recognized only sporadically and with low frequencies after vaccination, thus not allowing the definition of a vaccine-related reactivity pattern.

We did not test the samples after BNT162b2 administration, since autoantibody-mediated side-effects have been less frequently reported in RNA-based vaccines (27, 28).

Our data support the view that the immunization with the SARS-CoV-2 spike protein may trigger the production of low titer of aPL and other autoantibodies but does not affect the titer of pre-existing autoantibodies detectable in asymptomatic healthcare workers, ruling out the possibility of a widespread autoimmune humoral response shortly after vaccination.

On the other hand, there is evidence that autoimmune responses can take place in patients with acute COVID-19. In particular, autoantibodies were described against lung endothelial and epithelial cell surface antigens in patients suffering from SARS-CoV-1 disease (29). More recently, significant deposition of Ig and complement has been described in post-mortem biopsy specimens of various organs collected from COVID-19 patients but not in SARS-CoV-2 negative pathological controls (30). The lack of co-localization of the spike protein with the Ig and complement deposits was suggested to be the consequence of an auto-reactivity against self-antigens presented by the damaged tissues (30). Consistent with this hypothesis, two groups reported the new onset of autoantibodies against self-proteins in hospitalized COVID-19 patients and in healthcare workers with mild or asymptomatic infection (31, 32). It has been suggested that these autoantibodies may play a role in triggering and/or supporting the clinical manifestations of the disease. The lack of autoreactivity early after vaccination is not in clash with the abovementioned data, since active immunization is not associated with extensive inflammation and tissue damage as in the COVID-19, and the presentation of self-antigens in immunogenic forms is not likely to take place in the vaccinated subjects.

Autoimmune diseases are characterized by a long incubation period and the associated autoantibodies may be detectable even years before the clinical manifestations. We cannot rule out that autoimmune markers or even clinical autoimmune manifestations can develop over time, and the study has been planned to collect data and further biological samples in a longer follow-up (more than one year) taking advantage of the presence of the vaccinated subjects in our institutions.

## Data Availability Statement

The original contributions presented in the study are included in the article/**Supplementary Material**. Further inquiries can be directed to the corresponding author.

## Ethics Statement

The studies involving human participants were reviewed and approved by the Ethics Committee at Istituto Auxologico Italiano (08-01-2021). Vaxzevria samples were collected at IRCCS Ca’ Granda Ospedale Maggiore Policlinico in Milan (Polimmune-COVID: approved by Institutional Review Board Milano Area 2 and by the National Ethical Committee of Ospedale Spallanzani, #331_2020 and #665_2020). The patients/participants provided their written informed consent to participate in this study.

## Author Contributions

MOB, MB, RG, and PLM contributed to conception and design of the study and wrote the first draft of the manuscript. CB, PL, AGob, and ML performed the assays for autoantibodies detection, collected the results, and organized the database. ET, AD, IB, and FS provided the clinical data. AB and AGor evaluated the serological response to the vaccines. MB, AGob and FP performed the statistical analysis. GP and SA supervised the study. All authors contributed to manuscript revision, read, and approved the submitted version.

## Funding

The study was supported in part by Ricerca Corrente - Ministero della Salute, Italy 2020–2021 (to PM). This research was supported by the project COiMMUNITY (ID 1842163) funded by Regione Lombardia and co-funded under POR FESR 2014-2020 resources (to RG), and by the project COVID-2020-12371640 (to SA) funded by Ministero della Salute and by an unrestricted grant from Fondazione “Romeo ed Enrica Invernizzi.”

## Conflict of Interest

The authors declare that the research was conducted in the absence of any commercial or financial relationships that could be construed as a potential conflict of interest.

## Publisher’s Note

All claims expressed in this article are solely those of the authors and do not necessarily represent those of their affiliated organizations, or those of the publisher, the editors and the reviewers. Any product that may be evaluated in this article, or claim that may be made by its manufacturer, is not guaranteed or endorsed by the publisher.
