# Supplementary material for: Anti-Phospholipid Antibodies and Coronavirus Disease 2019: Vaccination Does Not Trigger Early Autoantibody Production in Healthcare Workers
Source: Front Immunol. 2022 Jul 15;13:930074. doi: 10.3389/fimmu.2022.930074 (PMC9334668; doi:10.3389/fimmu.2022.930074)
Supplement: Supplementary file 1 [file DataSheet_1.pdf]

**SUPPLEMENTARY TABLES**

**Supplementary Table 1.** Demographic characteristics of the vaccinated subjects.

| | **BNT162b2** | **ChAdOx1** |
|---|---|---|
| *Age* | 42±11 yrs | 38±9 yrs |
| *Gender (Female)* | 77/100 | 33/50 |
| *Familiarity for allergy/autoimmunity* | 16/100 | 1/50 |
| *Chronic diseases (incl. autoimmune disorders)* | 2/100 | 0/50 |
| *Chronic therapies (anti-hypertensive drugs)* | 3/100 | 0/50 |
| *Allergy* | 15/100 | 4/50 |
| *Infectious disease in the last 6 months (incl. COVID-19)* | 15/100 | 5/50 |
| *Other vaccinations in the last 6 months* | 35/100 | 0/50 |

**Supplementary Table 2:** Percentages of recorded side effects after vaccination.

| **Side effect** | **BNT162b2** | **ChAdOx1** |
|---|---|---|
| **Injection-site pain** | 63% | 68% |
| **Fatigue** | 23% | 28% |
| **Headache** | 12% | 15% |
| **Muscle pain** | 13% | 13% |
| **Fever** | 5% | 7% |
| **Joint pain** | 5% | 6% |
| **Chills** | 5% | 7% |
| **Nausea** | 4% | 5% |
| **Swelling** | 4% | 6% |

**Supplementary Table 3.** List of the 59 autoantigens specifically reacting with ChAdOx1 sera (at least one), selected by HuProt array analysis, as compared with HC sera.

| Gene symbol | Reviewed UniProt | *Gene name* |
|---|---|---|
| **AAK1_frag** | Q2M2I8 | *AP2 associated kinase 1* |
| **ADPRH** | P54922 | *ADP-ribosylarginine hydrolase* |
| **ANP32B** | Q92688 | *acidic nuclear phosphoprotein 32 family member B* |
| **APH1A** | Q96BI3-2 | *aph-1 homolog A, gamma-secretase subunit* |
| **C11orf84** | Q9BUA3 | *spindlin interactor and repressor of chromatin binding* |
| **C8orf37** | Q96NL8 | *chromosome 8 open reading frame 37* |
| **CD59** | P13987 | *CD59 molecule (CD59 blood group)* |
| **CFP** | P27918 | *complement factor properdin* |
| **CRABP2** | P29373 | *cellular retinoic acid binding protein 2* |
| **CTBP2** | P56545 | *C-terminal binding protein 2* |
| **DDX53** | Q86TM3 | *DEAD-box helicase 53* |
| **DEPP** | Q9NTK1 | *DEPP1 autophagy regulator* |
| **DNAJC12** | Q9UKB3-2 | *DnaJ heat shock protein family (Hsp40) member C12* |
| **DPCR1** | Q3MIW9-2 | *mucin like 3* |
| **ERP29** | P30040 | *endoplasmic reticulum protein 29* |
| **FAM107A** | O95990 | *family with sequence similarity 107 member A* |
| **FCHSD1** | Q86WN1-3 | *FCH and double SH3 domains 1* |
| **FKBP5** | Q13451 | *FKBP prolyl isomerase 5* |
| **GDI2** | P50395 | *GDP dissociation inhibitor 2* |
| **GSTT1** | P30711 | *glutathione S-transferase theta 1* |
| **HLCS** | P50747 | *holocarboxylase synthetase* |
| **HN1** | Q9UK76 | *Jupiter microtubule associated homolog 1* |
| **HSF2** | Q03933-2 | *heat shock transcription factor 2* |
| **INPP5B** | P32019 | *inositol polyphosphate-5-phosphatase B* |
| **IQWD1** | Q58WW2 | *DDB1 and CUL4 associated factor 6* |
| **KRTAP26-1** | Q6PEX3 | *keratin associated protein 26-1* |
| **LDOC1** | O95751 | *LDOC1 regulator of NFKB signaling* |
| **LGALS1** | P09382 | *galectin 1* |
| **LGALS3** | P17931 | *galectin 3* |
| **LGALS4** | P56470 | *galectin 4* |
| **LGALS9** | O00182-2 | *galectin 9* |

| | | |
|---|---|---|
| **LINC01465** | Q8N7H1 | *long intergenic non-protein coding RNA 1465* |
| **MED22** | Q15528-2 | *mediator complex subunit 22* |
| **MMADHC** | Q9H3L0 | *metabolism of cobalamin associated D* |
| **MRAP2** | Q96G30 | *melanocortin 2 receptor accessory protein 2* |
| **MRVI1** | Q9Y6F6-6 | *murine retrovirus integration site 1 homolog* |
| **MX1** | P20591 | *MX dynamin like GTPase 1* |
| **NCL** | P19338 | *nucleolin* |
| **NECAB2** | Q7Z6G3 | *N-terminal EF-hand calcium binding protein 2* |
| **OLIG2** | Q13516 | *oligodendrocyte transcription factor 2* |
| **PAFAH1B2** | P68402 | *platelet activating factor acetylhydrolase 1b catalytic subunit 2* |
| **PAFAH1B3** | Q15102 | *platelet activating factor acetylhydrolase 1b catalytic subunit 3* |
| **PEA15** | Q15121 | *proliferation and apoptosis adaptor protein 15* |
| **PFN1** | P07737 | *profilin 1* |
| **PGLS** | O95336 | *6-phosphogluconolactonase* |
| **PKNOX2** | Q96KN3 | *PBX/knotted 1 homeobox 2* |
| **RUNX1T1** | Q06455-4 | *RUNX1 partner transcriptional co-repressor 1* |
| **SEPSECS** | Q9HD40-3 | *Sep (O-phosphoserine) tRNA:Sec (selenocysteine) tRNA synthase* |
| **SH3BGRL** | O75368 | *SH3 domain binding glutamate rich protein like* |
| **SLCO4A1-AS1** | --- | *SLCO4A1 antisense RNA 1* |
| **SMRP1** | Q8NCR6 | *chromosome 9 open reading frame 24* |
| **TMOD1** | P28289 | *tropomodulin 1* |
| **TMOD4** | Q9NZQ9 | *tropomodulin 4* |
| **TOE1** | Q96GM8 | *target of EGR1, exonuclease* |
| **TPMT** | P51580 | *thiopurine S-methyltransferase* |
| **TUBB6** | Q9BUF5 | *tubulin beta 6 class V* |
| **UBQLN3** | Q9H347 | *ubiquilin 3* |
| **USO1** | O60763 | *USO1 vesicle transport factor* |
| **ZNRD1** | Q9P1U0 | *zinc ribbon domain containing 1* |
