# Supplementary material for: Anti-Phospholipid Antibodies and Coronavirus Disease 2019: Vaccination Does Not Trigger Early Autoantibody Production in Healthcare Workers
Source: Front Immunol. 2022 Jul 15;13:930074. doi: 10.3389/fimmu.2022.930074 (PMC9334668; doi:10.3389/fimmu.2022.930074)
Supplement: Supplementary file 2 [file DataSheet_2.pdf]

SUPPLEMENTARY FIGURES

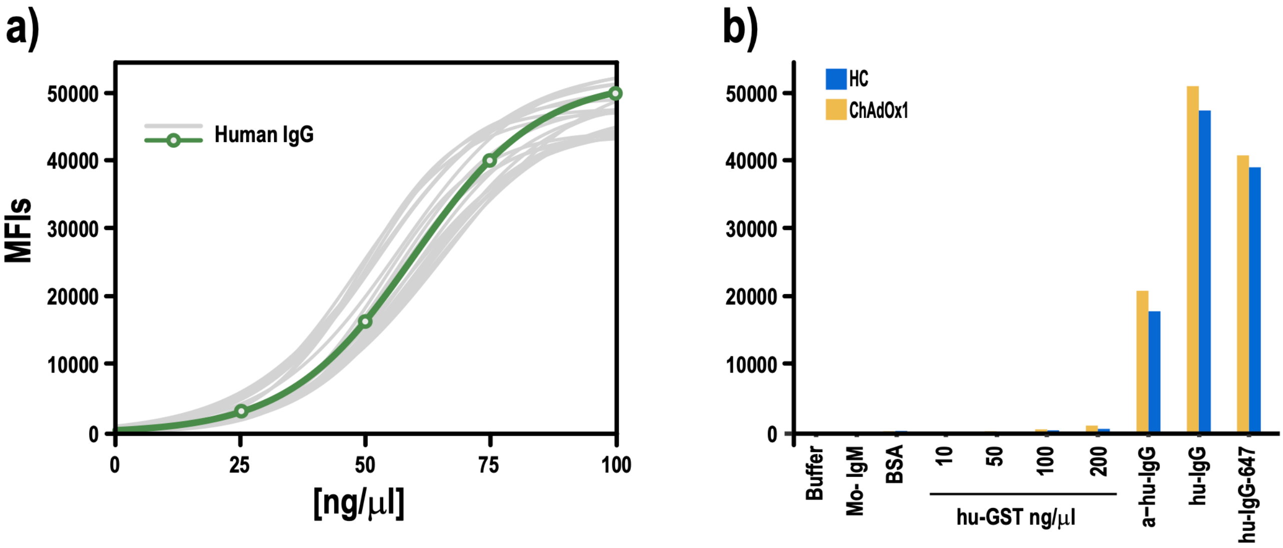

Figure S1: Correlation between MFI values and IgG concentration. A) Plots of human IgG curves used for protein array data normalization. In green a representative average curve. The dots correspond to the average MFI signal detected for each IgG concentration, while the solid line represents the interpolated resulting sigmoid curve. **B)** Bar-chart of representative control proteins into the arrays. Protein array MFI values of control proteins reacting with sera from vaccinated subject (ChAdOx1) and healthy controls (HC). In particular, MFI values of the following proteins are reported: Human Glutathione-S-Transferase (Hu-GST) curve, Bovine Serum Albumin (BSA), mouse immunoglobulin M (Mo-IgM), human immunoglobulin G (Hu-IgG), and Immunoglobulin 647fluor-conjugate.

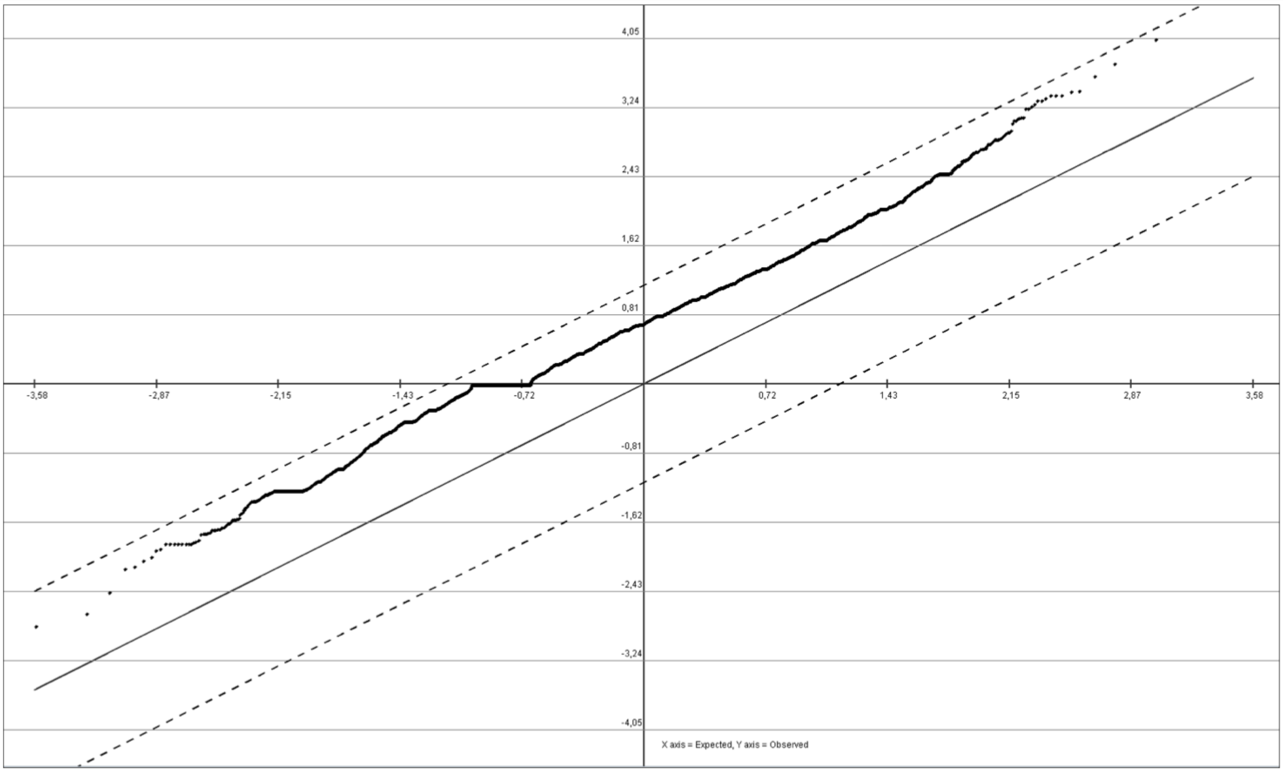

Figure S2. No antigens showed significant differential reactivity between vaccinated and HC. Scatter plot of the observed relative difference (d(i), *x* axes) versus the expected relative difference (dE(i), *y* axes). The solid line indicates the line for d(i) = dE(i), where the observed relative difference is identical to the expected relative difference. The dotted lines are drawn at a distance $\Delta = 1.15$ from the solid line. Imputation was conducted by K-Nearest Neighbors for a number permutation equal to 100.

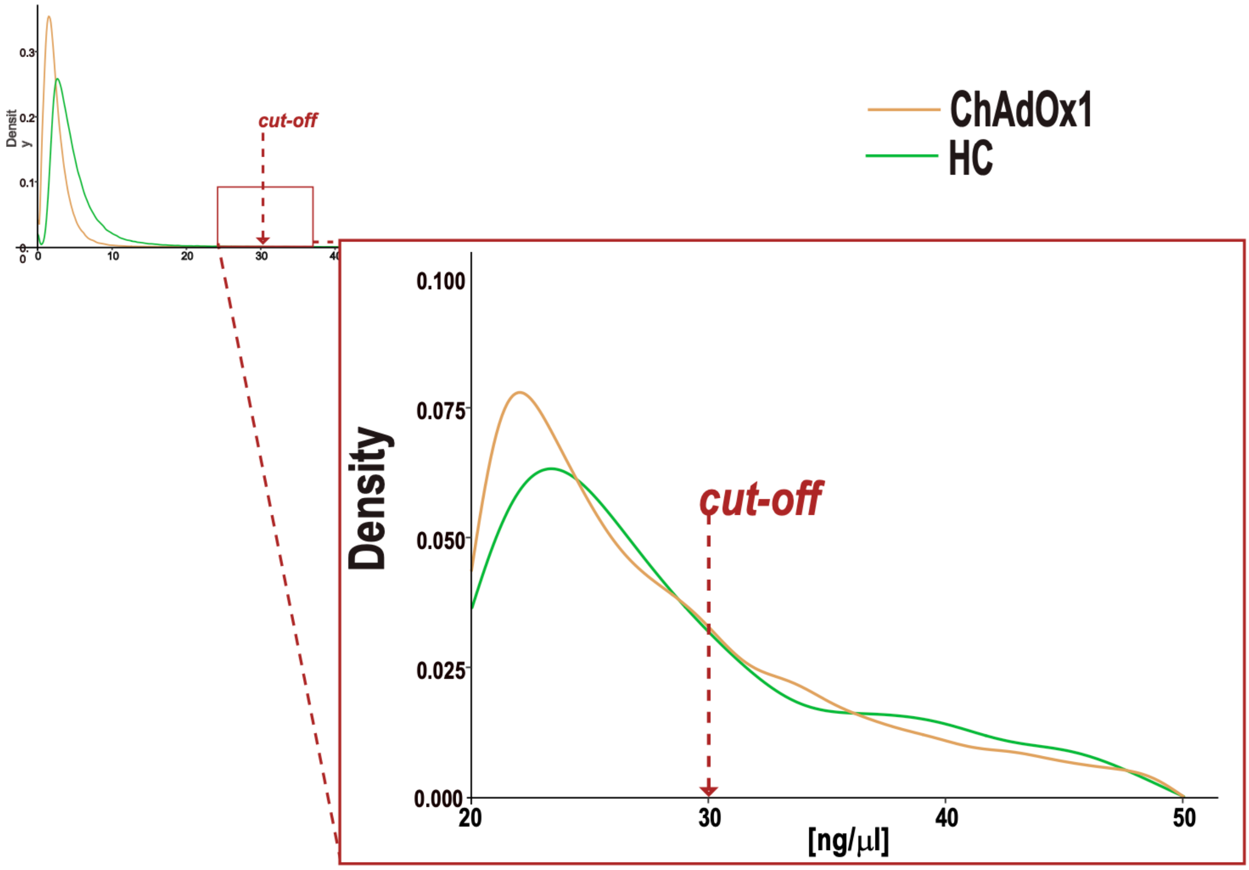

Figure S3: Graphic representation of distribution of immune-reactivity of ChAdOx1 subject and HC on the protein array. In order to establish a threshold to select potential features, we analyzed the protein reactivity distribution analysis of ChAdOx1 and HC sera. Cut-off: Concentration > 30 was used as threshold. *x*-axes indicate values for concentration per protein, while *y*-axes are unit relative frequencies.
